# Second Clinical Report on Continued Fever, Based on an Analysis of Forty-Eight Cases

**Published:** 1851-08

**Authors:** Austin Flint

**Affiliations:** Prof. of the Principles and Practice of Medicine, and Clinical Medicine, in the University of Buffalo


ART. V.—*Second Clinical Report on Continued Fever, based on an analysis of forty-eight cases.* By AUSTIN FLINT, M. D., Prof. of the Principles and Practice of Medicine, and Clinical Medicine, in the University of Buffalo.

(*Continued from page* 80.)

SECTION THIRD.

*Symptoms referdble to the general aspect and expression of countenance.*

[No. for Sept., 1850, page 193.]

The complexion, or color, of the face, was almost uniformly altered from that of health. Redness, due to capillary congestion, was present in *twenty-six* of the *twenty-nine* cases of *Typhoid*, and in the remaining *three* cases its absence is not affirmed. Its presence was noted in *all* of the *ten* cases of *Typhus.* The redness now referred to, is that described in the first Report, resembling the appearance of the surface after exposure to cold. It is not a *flush*, like that occasioned by mental emotions, or occurring in connection with excitement of the circulation by exercise, or with high febrile movement. The latter is frequently, if not generally, observed in the early part of the career of Continued Fever, and is noted in some of the histories in this collection; but the *congestive redness*, as it is called in the records, is evidently owing to a passive accumulation in the capillary vessels. The color is more dull than when the face is flushed, and the temperature is but little, if at all, perceptibly raised. It is especially marked on the cheeks, and is sometimes confined to the cheeks, but without being circumscribed by a sharp border.

The redness differs, in different cases, in degree. Of the *twenty-six* cases of *Typhoid* in which it was more or less present, it was characterized as *intense* in but a single case; it was *slight* in *six* cases, and in *eighteen* cases it was either *moderate*, or *considerable.* In the cases of *Typhus*, the redness was *intense* in *five* cases, and in no case was it but *slight.* In proportion to the intensity of the congestion, the surface presented a dusky or dingy hue.

The disparity between the two types of the Fever, as respects this symptom, is a distinctive point worthy of note. The redness is rarely intense in *Typhoid*, although more or less present in nearly all cases; but in *Typhus* it would seem to be uniformly present, and is intense in a much larger proportion of cases. A dusky or dingy color of the face is, thus, somewhat distinctive of the latter type.

A symptom so constantly associated with the disease, although not of a very striking character, must have some pathological significance, especially if it be not a symptom common to other affections. Of the extent to which it is associated with different diseases. I cannot speak from an adequate number of recorded observations, but all observers are aware that it occurs in connection with affections involving obstruction of the general circulation. Thus it it present in diseases of the heart, in pneumonitis, pleuritis with large effusion, etc. The symptom, under these circumstances, is induced purely by physical causes of an obvious character. That the symptom is not due to these causes in Continued Fever, is rendered probable by the facts presented in the former Report. These facts show that it does not sustain relations with symptoms referable to the heart and lungs, which should exist if it were incident to morbid conditions of these organs mechanically obstructing the general circulation. The reader is referred to the first Report for these facts. A fair inference therefrom is, that the capillary congestion arises from an obstacle inherent in the capillary system. And if, as is probable, this obstacle pertains to the blood itself, rather than to the capillary vessels, the symptom under consideration is to be regarded as favoring the humoral doctrine of the pathology of Continued Fever.

The face is not the only seat of capillary congestion in Continued Fever. The same condition generally obtains, more or less, over the surface of the body, as will be seen in the section devoted to the symptoms referable to the skin. It is always present on the face, if it exist in other portions of the cutaneous system, and is invariably more marked in the former situation. It is sometimes to be observed on the face when it is not obvious elsewhere.

It is of importance to bear in mind that the same condition of the capillary system, it is probable, obtains in internal organs. This may be presumed, especially if the source of the congestion is considered to be in the blood itself.

Congestive redness of the face was usually apparent from the time the patients came under observation. It continued generally through the greater part of the frebrile career, varying somewhat in degree. In some cases it persisted after convalescence was declared, but in other cases it gradually diminished and disappeared on, or shortly before convalescence.

A *circumscribed flush* of the cheeks was noted in several cases, viz; in *five* cases of *Typhoid*, in *two* cases of *Typhus*, and in *two* cases of *Doubtful type*, in all, *ten* cases. This symptom, which is somewhat characteristic of primitive pneumonitis, appears to be significent of the same affection occurring as a complication of fever. Of the *five Typhoid* cases in which it was present, pneumonitis undoubtedly co-existed in *four*, and probably in the *two* remaining cases. Of the *two Typhus* cases, pneumonitis was present in *one*, and probably present in the *other*. The *two* cases of *Doubtful Type* were complicated with pneumonitis.

The expression of countenance, as indicative of the condition of the mind, was almost invariably more or less modified. The terms used to denote the appearances in this respect are, *vacant*, *dull*, *heavy*, *listless*, *indifferent*. In *one* case, characterized by violent delirium, the expression indicated terror. *Brightness* of expression was observed to accompany improvement in other symptoms. A decided change for the better was frequently revealed by a glance at the countenance of the patient. Patients affected with fever, almost invariably present, during the febrile career, a grave, sedate expression, and a smile on approaching the bed side, is often the harbinger of convalescence. There are some exceptions to this remark. In a few cases embraced in the first collection, the countenance throughout the febrile career wore an aspect of mirthfulness and good humor, the patients presenting, on convalescence, a more serious expression, thus reversing the general rule. This was true of *one* of the cases of the *Typhus* type in the present collection.

### SECTION FOURTH.

*Symptoms referable to the Nervous System, Mind, Sleep, Coma, Senses and Sensibility, Muscular Contractions, etc.* [No. for Sept. 1850, page 199.]

*Delirium* was present in a large proportion of the cases. Excluding the cases in the histories of which the facts with respect to this symptom were not recorded, of the remaining *thirty-eight* cases more or less delirium existed in *thirty*. The proportion of cases of the two types, *Typhus* and *Typhoid*, in which the symptom was present, is as follows:—of *twenty-two* cases of *Typhoid*, of which the histories contain information on this point, it existed in *seventeen*. Of *nine* cases of *Typhus*, it existed in *eight*. It existed in *five* of *seven* cases of *Doubtful type*.

It is interesting to see how nearly the above results approximate to those developed by the analysis of the first collection of Cases. The proportion of cases in which delirium was present in the two collections respectively, is as

30–38 is to 43–52. Comparing the two types, *Typhus* and *Typhoid*, in the two collections, with respect to this symptom, in *Typhoid* the relation is as 17–22 is to 23–30; in *Typhus*, as 8–9 is to 11–12; in the cases of *Doubtful Type*, as 5–7 is to 7–8. It is hardly to be supposed that the close approach to equality in these results, is due simply to accidental coincidence, and, therefore, it may be inferred that these results express laws of the disease as respects the numerical ratio in which, in a given number of cases of either type of the fever, this symptom may be expected to be present.

It will be observed in this, as in the former collection, that delirium is present in a much larger proportion of cases of the *Typhus*, than of the *Typhoid* type.

In the great majority of the cases in which more or less delirium existed, it was passive in its character, that is, the patients were not violent, so as to require restraint. Great activity, or violence, characterized the delirium in only *one* case of *Typhoid*, and in this case it was a very prominent and persistent trait. In *two* cases of *Typhus* it assumed somewhat an active character, for a short time, but much less in degree than in the *Typhoid* case just mentioned. The cases varied considerable as respects the duration and degree of the delirium, as well as the period in the febrile career at which it appeared. It became developed earlier in the course of the disease, as well as more constantly, in the cases of *Typhus*, than in the cases of *Typhoid*, In some cases it continued through the greater part of the febrile career; in other cases it was of short continuance. It was only observed, in some cases during the latter part of the career; in other instances it appeared at an earlier date, and ceased some days before convalescence. Occasionally it occurred at irregular intervals during the disease. It was almost invariably more marked during the night, than during the day time, and frequently existed only at night.

The manifestations of delirium consisted in incoherent talking, and efforts to get out of bed, in some cases the former only, and in other cases both conjoined. The hysterical character described in the former Report as occurring in a few cases, was not observed in any of the cases in the present collection.

In nearly all the cases in which more or less delirium was not present, or not apparent, the fever was of a mild grade of intensity.

In the fatal cases the facts with respect to delirium are as follows:—Its presence was noted in the histories of all the fatal *Typhoid* cases save two, i. e. in *five* of *seven* cases. Of the two cases in which its presence is not noted, in *one* the patient died from intestinal perforation, an event which has been found to be more likely to occur when the disease is mild, than when it is severe. In the *other* case the patient died on the second day after admission, in apoplectic coma. Of the *five Typhus* cases in which it was present, it was a prominent symptom in *all* save *one*. In the history of the *single* excepted case, it is distinctly noted that no manifestations of delirium were observed. This exception is important, especially when it is considered that the excepted case was the only one in the *Typhus* collection in the history of which the absence of delirium is noted. The fact suffices to show that absence of delirium does not, in itself, justify a favorable prognosis, while the burthen of facts undoubtedly favors the conclusion that, generally, this symptom denotes a certain degree of gravity in the disease, and that, being a prominent feature in a larger proportion of fatal cases than in those ending in recovery, it should influence, in proportion to its prominence, unfavorably, the prognosis.

The delirium, in Continued Fever, in the great majority of cases, is passive, the patients remaining quiet, but talking irrelevantly, and incoherently; or, in addition thereto, making efforts to get up, but easily persuaded, for the time, to desist, or return to the bed. Sometimes, but this is rare, the delirium is noisy, the patient vociferating, or shouting; and occasionally some difficulty is experienced in persuading him to keep in bed. In a small proportion of cases the delirium is remarkably persistent and violent, constituting the most prominent feature of the disease. *Three* instances of the latter description are embraced in the one hundred cases I have analyzed. *Two* of these are included in the first collection. The delirium, in these cases, commenced rather early, and continued without cessation, or abatement, up to a few hours before death. The manifestations consisted in loud talking and incessant attempts to get out of bed, rendering considerable restraining force, and constant care, on the part of the attendants, necessary. Both cases were fatal. Both were of the *Typhoid* type. These cases are distinguished, not by the occurrence of active delirium, for this was true of a few other cases, but by its steady continuance, as well as violence. It is important to be aware of the fact that Continued Fever may be characterized by an excessive predominence of this symptom. Without this knowledge, and due care in the diagnosis, the disease may be thought either to consist in, or to involve encephalic inflammation — an error of some practical moment; or, more than this, it may be considered to be merely a functional affection of the brain — mania. The reports of superintendents of insane asylums, state that such cases are occasionally sent to these institutions, the patients being thereby exposed to the injury of a journey thither, as well as having failed to receive the treatment which a correct appreciation of the disease might have suggested.

In illustration of the class of cases of which I have been speaking, a summary of the more important points in the history of the single case in the present collection may be interesting.

The patient was a German laborer, aged 27 years. The case was one of those in which the disease was attributed to contagion. Four weeks before being attacked, he had taken charge, at night, of a brother affected with fever. He had slept with him, and labored, as usual, during the day. He was seized with pain in the head, etc., about ten days before entering the hospital. Four days before his admission, he got bled on account of the cephalalgia. The next day he became actively delirious. He had no medical attendance prior to his entering the hospital. These facts pertaining to the previous history were not ascertained until after the decease of the patient. He spoke no English, and, moreover, was incompetent, from the condition of his mind, to give any account of himself. From the resemblance of the delirium to that in the two cases previously observed, and from the associated symptoms, I had no difficulty in arri\ing at the diagnosis. On his entrance, Oct. 28th, he was actively delirious, constantly talking, and endea oring to get out of bed. Head was hot, skin warm, tongue (which could but imperfectly be examined) appeared to be dry and hard. Face presented considerable congestive redness, which was also apparent on the upper extremities. Pulse 120, and moderately developed. Abdomen meteorized. He was apparently quite deaf. Did not reply to questions. Eyes not injected.

I had resolved, after observing the two former cases of this variety of the disease, if another instance should ever present itself, to resort to opiates freely, in conjunction with antimony. I accordingly prescribed, in this case, on the first day, S. morphiæ, gr. 1-4 hourly, which was continued until one and a half grains had been taken. Tartrate of antimony was given in doses of gr. 1-8 every half hour, and continued for six hours. This was at evening, when he had just entered. At 10 1-2 P. M., he was sleeping quietly, and he continued to sleep a portion of the night.

On the 29th, the delirium, at morning, was moderate; pulse 108; respirations 18; meteorism, and tenderness in right iliac region; tongue dry and fissured, protruded slowly and incompletely; one copious dejection, not involuntary. The directions for treatment were to apply cold to the head, an emp. vesicat. to nucha, and, if the delirium increased, to resort to morphia and antimony, repeated at short intervals until he became quiet.

30th. The record for this day, made by me, is as follows: — "Passed a wakeful night, with constant, active delirium. His efforts to get out of bed (except during night before last, when he obtained some sleep) have been incessant. He requires constant watching, and occasionally restraint. The expression of the face denotes apprehension, suspicion, and a fixed purpose. Judging from his countenance, and efforts, his impulse is to escape from a situation of imaginary danger. Sometimes he does not reply to questions; sometimes he replies coherently, and sometimes incoherently. He seldom speaks of his own accord, but occasionally talks with a loud voice.

The tongue is dry and hard, and partially protruded, after being requested several times. One copious dejection. Abdomen tympanitic. Perspiration stands in drops on the face, and is moderate over the body and extremities. Pulse 128, small, and feeble. Respirations, 32. Complains of pain in the head. Had passed no urine, and catheter was introduced, drawing off about thirty-two ounces."

During this day, 2 1-2 grains of morphia were administered in hourly doses of gr. ss. At 4 1-2 P. M., he was sleeping, and had been quiet for nearly two hours. Respirations, 24, *inspirations shortened and quickened.* Perspiration in drops on the face. Pulse 148, very small and feeble. Abdomen tympanitic. The treatment directed was, morphia, *pro re nata;* camphor, grs. iis, dissolved in chloroform, hourly; brandy, ℥*ss.* hourly. He was somnolent most of the night, and died at 7, A. M., the following morning.

The autopsy, performed twelve hours after death, disclosed the characteristic lesions of Peyer's glands, and the mesenteric ganglions; slight subarachnoid effusion; about an ounce of serum (estimated) in the arachnoid cavity; moderate congestion of brain; no exudation of fibrin or opacity of arachnoid; cerebral substance normal.

In two of the three cases resembling each other in the predominance of active persistent delirium, post mortem examinations were made. The appearances in the case in the first collection which was examined, are given in the former Report. In neither were there any appreciable evidences of encephalic inflammation. Indeed, the symptoms are insufficient to sustain the supposition that the delirium is due, in such cases, to an inflammatory condition. The phenomena which are associated with active delirium in encephalitis are not present. In each of the three cases bleeding early in the disease was prescribed, but without any relief. The free use of morphia, although it succeeded in overcoming, for a time, the delirium, and inducing quietude and somnolency, exerted no favorable effect upon the issue of the disease.

It is to be observed, that the three cases ended in death. So far as my experience goes, therefore, persistent, violent delirium is to be regarded as a fatal symptom.

The fact should be noted that the case just detailed was of the *Typhoid* type. The three cases were of this type.

In the former Report a few remarks were offered in connection with the inquiry, "upon what pathological condition, or conditions, is delirium in Continued Fever dependent?" Observations have abundantly established, that it is not due to inflammation, and hence, the occurrence of encephalitis, as a complication of the fever, is not to be predicated upon this symptom, however prominent it may be. This is an important practical fact. Is it not frequently overlooked? That this symptom is incident to the morbid condition, whatever it be, which is the essence of Continued Fever, appears to be, as intimated in the former Report, a fair conclusion, in accordance with what may be enunciated as a rule of pathological reasoning, viz., that whenever any symptom, in a large proportion of cases, is observed to be associated with a disease, and found, by a proper analysis of cases, not to be dependent on any of the other appreciable events entering into the history of the disease, it is to be considered as sustaining a direct relation with that which constitutes the *proximate cause* of the disease. If, for example, Continued Fever involves, as its prime, essential morbid condition, a special morbid change in the blood, the delirium, so frequently incident to the progress of the disease, is a result of the circulation of this morbid blood within the brain, and it would be expected, what, as a general rule, is true, that the degree of delirium should correspond with the gravity of the disease.

As regards the mental conditions, irrespective of delirium, in this, as in the former collection of cases, *hebetude* was the prevailing trait. The mind appeared to act slowly and with more or less difficulty. The patients, when awake, took but little notice of what was passing around them; their replies to questions were short; they seldom seemed to manifest a disposition to describe or explain their sensations, and very rarely showed any anxiety as to the issue of the disease. There were considerable variations in the degree of indifference and sluggishness, and, as a general remark, it obtained earlier, and was more marked in cases of *Typhus*, than in those of *Typhoid*. Promptness in comprehending and replying to questions; an increased disposition to make inquiries, to indicate wants, and a returning appreciation of their situation, are among the more obvious of the indications of a favorable change in the progress of the disease.

The statements of fever patients with respect to the events that transpire in the case, from day to day, are not to be relied upon. Their replies to questions may be sufficiently reasonable, but quite the reverse of the truth. For examples, they may say that they have slept well, when the whole night has been passed in busy delirium, and vice versa; they may say they have had dejections from the bowels, when none have occurred, etc. They frequently appear to reply to questions without any reflection, and entirely at hazard. But even if they are stimulated to direct their thoughts to any point, the recollection is apparently unable to discriminate between what has really occurred, and the ideas that have passed through the mind. The memory may be tested by asking the time of the day, the day of the week, when the preceding visit was made, etc. Patients are often wholly unable to answer these questions correctly. It is therefore obvious that histories of cases made up of the statements of patients, would be likely to contain not a little that was erroneous.

*Sleep.*— A disposition to somnolency was noted in several of the histories, viz., in *thirteen* cases of *Typhoid*, and in *three* cases of *Typhus*. The proportion of cases in which this symptom appears, is considerably less than in the former collection, and, inasmuch as it is seldom stated that the symptom was not present, I am led to think that it may not have been noted save in the cases in which it was more especially marked. In all but *one* case the patients were easily roused, but lapsing into the somnolent state directly afterward. In *five* of the *seven* fatal cases of *Typhoid*, this symptom was present, and, of the *two* excepted cases, in *one* active delirium existed, and in the other case the patient died in apoplectic coma the day after admission. Judging by the results of the present analysis, it would seem that this symptom indicates a severity of disease greater than the average, and this accords with the results obtained by Louis and Jackson.

It is not to be understood that in the cases in which this symptom was present, it continued throughout the febrile career. It was present more or less, in some cases only of temporary duration, and sometimes observed at different periods in the same case.

In *nine* of the *thirteen Typhoid* cases characterized by somnolency, delirium, to a greater or less extent, entered into the history. Patients were sometimes somnolent during the day time, with slight, if any manifestations of delirium, and at night talkative, and attempting to get out of bed.

Delirium, hebetude, and somnolency, which were thus frequently exhibited almost simultaneously in the same case, are probably dependent on a similar morbid condition, each denoting an enfeebled state of the cerebral organs. At the first blush this explanation may not appear to be applicable to delirium, but it will be found to be the most rational view, on a little reflection. The mental aberration in the great majority of cases has been seen to be passive; the manifestations do not display increased energy of mental action, but the patient talks and acts under the influence of dreamy delusions, somewhat as when the brain is oppressed by intoxication carried beyond the bounds of excitement. These delusions are generally not persistent, but constantly varying. The mind has not sufficient strength to preserve the same train of ideas. Hence the incoherent muttering which generally characterizes the delirium. It may be supposed that in the few cases in which the delirium is said to be active or violent, that there must be increased functional activity of the brain, and that these are exceptions to the general rule. The most rational mode of accounting for the character of the delirium in these cases, is, that the insane delusions are of a character to rouse energetic expressions and actions by exciting sensations of apprehension and terror. This being the case, the vigorous and continued efforts of the patient to resist restraint, do not denote increase of nervous power, more than in *delirium tremens*, to which affection, the mental condition in fever referred to bears, a strong analogy.

The term somnolency, in the foregoing remarks, it will be understood, of course, is not intended to be applied to veritable sleep, but to that state, sometimes called *coma-vigil*, in which the patient lies in a semi-unconscious state, apparently, but not in reality sleeping, from which, usually, he is readily roused, and to which he relapses so soon as the efforts to rouse him are discontinued. With respect to true sleep, the patients were daily asked whether they had slept much, or little, during the night, and their replies recorded. Very little importance, however, is to be attached to the information thus obtained. Patients oftener reported that they had slept well than the reverse, and they frequently made this report when the night had been, in reality, much disturbed. Sometimes want of sleep, and inability to sleep, were the subjects of complaint. This was more apt to be the case when the disease was comparatively mild, and the nights were passed more quietly, and apparently more comfortably, than in other instances in which no such complaints were made; the explanation of which probably is, that in such instances the powers of the mind are less compromised, and the patients are able to appreciate causes of discomfort, which, in graver forms of the disease, although existing to a greater extent, do not produce as much conscious impressions, nor are the impressions so well retained in the memory owing to weakness of this retentive faculty.

*Coma.*—Sudden, or apoplectic coma is an event which occasionally becomes developed in the progress of Continued Fever. It occurred in *four* of the cases first analyzed, but in *one* case only in the present collection, the case ending fatally. In *two* other cases there was an approach to the sudden development of the comatose state.

The following is a condensed history of the case in the present collection characterized by the occurrence of complete and fatal coma: — Patrick Enright, Irish, laborer, of good constitution, aged 38, single, entered hospital at noon, Dec. 10. He was attacked four or five days previously, with pains in head, loins, and limbs, which still continue. Has had loss of appetite, great thirst, some nausea and vomiting, bowels moved daily, complained of vigilance. He did not present, on his entrance, high febrile movement; pulse not much accelerated, and was well developed. Tongue moist and coated. Skin warm, and rather dry. At evening he appeared comfortable.

At 1, A. M., the next day, he was found in the following condition, viz. face and upper extremeties covered with cold clammy perspiration; body and lower extremities warm; pulse small and feeble, and accelerated; unconscious; respiration irregular, labored, with puffing of the cheeks in expiration; froth exuding from mouth; difficulty of deglutition.

Sinapisms were applied to extremities, and to nucha; friction with dry mustard to arms; brandy and ammonia, internally, freely administered.

Some improvement in the symptoms followed, which was but temporary, and death occurred at 7, A. M.

The morbid appearances within the brain, fourteen hours after death, consisted in considerable congestion of the veins of the superficies; moderate increase of subarachnoid effusion, and about an ounce of serum in the arachnoid cavity. The characteristic intestinal lesions of *Typhoid* fever were present in a marked degree. The kidneys presented some degree of granular degeneration.

It is not easy from these appearances to deduce the explanation of the sudden coma and death. My own impression is, that the symptoms and termination are most rationally accounted for on the supposition of effusion into the arachnoid cavity, as the result, probably, of vascular engorgment, and perhaps a morbid condition of the blood itself. Under this supposition, mechanical compression of the medulla oblongata was the cause of death, and the preceding phenomena. To this lesion, as one liable to occur, and occasion a fatal termination in some cases in which the loss of consciousness, etc., are not developed so suddenly as to constitute apoplectic coma, I shall refer in other connections.

Of the two cases mentioned in which there was an approach to a condition similar to that in the case just given, some account may possess interest. Both cases are included among those of *Doubtful type*. In one case the patient entered on the sixth day after the first symptoms of illness. There were no indications of unusual severity of disease. The day after his admission, in the morning the symptoms were as follows: — face and hands moderately congested; skin warm and dry; tongue dry and coated, of a dark color; some appetite; much thirst; no dejection, no abdominal tenderness; pulse 104, tolerably developed; no cough, respirations 16; incoherent talking during night; some carphologia this morning; restless, frequently changing his position.

At 6, P. M., copious perspiration, extremities cold and clammy; pulse 140, small and feeble; respirations irregular, catching, and he was supposed to be moribund. Sinapisms were applied, a blister to the nucha, and stimulants (brandy and ammonia) freely administered. Snow was applied to the head. He began to improve at about 10, P. M., and on the day following, the record states that he was rational, skin warm and moist, pulse 88, respirations 12 and regular, etc. Convalescence was soon established.

In the second case, the patient entered on the sixth day after the first symptoms of illness, and presented the symptoms of fever of a moderate grade of intensity. On the evening of the day of his admission, the record states that the respiration became much embarrassed, perspiration copious, and he was nearly comatose. The symptoms more in detail, and the therapeutical measures pursued, are not stated. On the following day he was quite comfortable, pulse 72, skin warm and moist, respirations 16, etc. He reported that he slept well during the night. Convalescence, in this case, was not declared until eight days after admission.

In these two cases there seemed to be an approximation to that condition, suddenly induced, which, in the case previously given, ended in fatal coma. The pathological explanation of the latter is probably measurably applicable to the former. The condition, whatever it be, seems to be analogous to that which in some of the cases was developed more gradually, determining the mode of dying. To these cases reference will be made hereafter.

(*To be continued.*)

